# Integrated transcriptomic and proteomic profiling implicates prostaglandin–nitric oxide network dysregulation in uterine microcirculatory impairment in primary dysmenorrhea

**DOI:** 10.3389/fimmu.2026.1833346

**Published:** 2026-05-11

**Authors:** Yufei Li, Zongtong Yang, Beibei Yu, Xinjun Zhang, Xiaojing Li, Wenjing Chen, Mengyu Yuan, Zaiyun Sui, Yue Zhang, Wenjing Hou, Demin Gao

**Affiliations:** 1 School of Pharmacy, Shandong University of Traditional Chinese Medicine (TCM), Jinan, China; 2 Department of Anesthesiology, the First Affiliated Hospital of Shandong First Medical University & Shandong Provincial Qianfoshan Hospital; Shandong Institute of Anesthesia and Respiratory Critical Medicine; Shandong Provincial Clinical Research Center for Anesthesiology, Jinan, China; 3 Shandong Academy of Chinese Medicine, Jinan, China; 4 Jining Medical University, Jining, China; 5 Qilu Institute of Technology, Jinan, China

**Keywords:** nitric oxide, primary dysmenorrhea, prostaglandin, proteomics, transcriptomics, uterine microcirculation

## Abstract

**Background:**

Primary dysmenorrhea (PD) is a prevalent gynecological disorder characterized by severe menstrual pain. Although excessive prostaglandin activity is a recognized driver of uterine hypercontractility, the molecular mechanisms linking prostaglandin imbalance to nitric oxide (NO) deficiency, coagulation abnormalities, and impaired uterine microcirculation remain incompletely understood.

**Methods:**

A PD model was established in 12 female Sprague–Dawley rats (control, n = 6; PD, n = 6) using estradiol valerate, repeated cold exposure, and oxytocin stimulation. Behavioral testing, biochemical and hormonal assays, histopathological evaluation, coagulation analysis, uterine microcirculation assessment, integrated transcriptomic and proteomic analyses, and targeted measurement of arginine and proline were performed to characterize PD-associated alterations.

**Results:**

PD rats exhibited marked hyperalgesia, uterine hypercontractility, and reduced uterine blood perfusion. These changes were accompanied by significantly elevated uterine PGF_2_α and PGE_2_ levels, together with an increased PGF_2_α/PGE_2_ ratio (*P* < 0.01), indicating a shift toward a contractile and vasoconstrictive prostaglandin profile. PD rats also showed decreased plasma NO and β-endorphin (β-EP) levels, altered estradiol (E_2_) and progesterone (P) levels, uterine histopathological injury, and coagulation disturbance, consistent with impaired vascular regulation and endogenous analgesic capacity. Integrated transcriptomic and proteomic analyses revealed widespread molecular dysregulation in PD, with arginine and proline metabolism identified as the only pathway significantly enriched at both levels. Consistently, targeted measurement confirmed elevated uterine arginine and proline levels, suggesting a potential impairment in arginine utilization that may contribute to reduced NO bioavailability.

**Conclusion:**

These findings support a systems-level model in which prostaglandin imbalance, coagulation-associated microcirculatory dysfunction, and dysregulated arginine–proline metabolism may jointly contribute to uterine ischemia and pain sensitization in PD. Our study proposes a refined molecular framework for PD pathogenesis and highlights the arginine–NO axis as a potential therapeutic target tworthy of future investigation.

## 1 Introduction

Dysmenorrhea represents a substantial global health burden, with a recent systematic review and meta-analysis of 336 studies from 70 countries and 3,245,020 women estimating a pooled prevalence of 71.3% (95% CI, 68.7%–73.8%) worldwide and 73.0% (95% CI, 68.0%–78.0%) for primary dysmenorrhea (1). Clinically, PD is defined as cyclic lower abdominal pain occurring during menstruation in the absence of identifiable organic pelvic disease (2). Severe cases are frequently accompanied by nausea, vomiting, fatigue, anxiety, and depression, significantly impairing quality of life and work productivity (3).

From a pathophysiological perspective, PD has traditionally been linked to excessive production of uterine cyclooxygenase-derived mediators, particularly prostaglandins such as PGF_2_α and PGE_2_, as well as other bioactive lipid mediators including thromboxane, which together promote abnormal uterine hypercontractility, spiral arteriolar vasoconstriction, and subsequent uterine ischemia and hypoxia (4). In addition to cyclooxygenase-derived products, other biomolecular pathways, including leukotriene signaling, vasopressin-related uterine activity, inflammatory responses, oxidative stress, and neuroendocrine dysregulation, have also been implicated in PD pathophysiology (5). These interacting processes activate nociceptive afferent fibers and ultimately contribute to menstrual pain. However, increasing evidence suggests that PD is not solely a prostaglandin-driven disorder, but rather a multifactorial condition involving inflammatory signaling, oxidative stress, neuroendocrine imbalance, and microvascular dysfunction (6, 7).

Nitric oxide (NO) is a critical endogenous vasodilator that plays an essential role in regulating uterine blood flow, smooth muscle relaxation, and pain perception (8, 9). Reduced NO bioavailability has been reported in PD patients and experimental models, yet the upstream metabolic and molecular mechanisms responsible for NO deficiency remain unclear (10). Notably, NO synthesis is tightly regulated by arginine metabolism via NO synthase (NOS), which is sensitive to inflammatory cytokines, oxidative stress, and hormonal fluctuations (11, 12). Therefore, perturbations in amino acid metabolism may represent an underappreciated contributor to PD pathogenesis.

From the perspective of traditional Chinese medicine, PD, particularly the cold coagulation and blood stasis subtype, is characterized by impaired blood circulation, cold-induced vasoconstriction, and stagnation of qi and blood within the uterus (13). This conceptual framework aligns closely with modern observations of microcirculatory dysfunction, coagulation abnormalities, and other pathological alterations in PD.

High-throughput omics technologies, including transcriptomics and proteomics, provide powerful tools for exploring disease mechanisms at a systems level (14, 15). While individual omics approaches have been applied to PD research, integrated transcriptomic and proteomic analyses remain scarce. Importantly, combined transcriptomic and proteomic profiling facilitates the identification of convergent regulatory pathways that are consistently altered at both transcriptional and translational levels, thereby improving biological interpretability (16).

In this study, we established a well-characterized rat model of PD with cold coagulation and blood stasis syndrome and performed integrated transcriptomic and proteomic analyses of uterine tissue, together with targeted measurement of arginine and proline. We aimed to (i) comprehensively characterize the behavioral, biochemical, vascular, and pathological features of PD; (ii) identify key molecular pathways involved in PD pathogenesis; and (iii) explore the potential role of arginine–NO metabolic dysregulation in vascular dysfunction and pain in PD.

## 2 Materials and methods

### 2.1 Animals and ethical approval

All experimental procedures were approved by the Institutional Animal Care and Use Committee of Shandong Academy of Chinese Medicine (Approval No. SDZYY20210925001). Female Sprague-Dawley rats (8 weeks old, 210 ± 10 g) were obtained from Jinan Pengyue Laboratory Animal Breeding Co., Ltd. Animals were housed under standard conditions (22-25 °C, 50-60% humidity, 12 h light/dark cycle) with free access to food and water.

### 2.2 Sample size determination

The sample size was determined with reference to previous studies using similar rat models of primary dysmenorrhea and based on practical and ethical considerations to minimize animal use (17). No formal *a priori* power analysis was performed.

### 2.3 Establishment of the primary dysmenorrhea model

**Figure 1:**
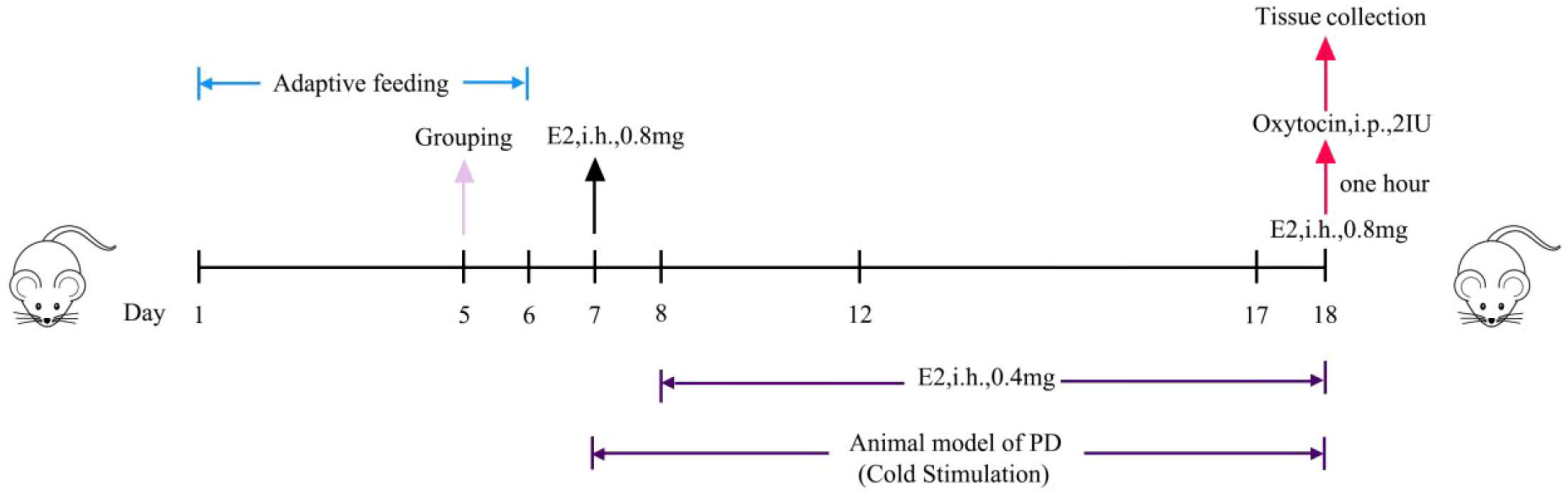
Schematic diagram of the animal treatment procedure. i.h., hypodermic injection; i.p., intraperitoneal injection.

Rats were acclimatized for 7 days and randomly assigned to a control group (CG, n = 6) or PD model group (PDG, n = 6). The PD model was established using a combination of hormonal stimulation, repeated cold exposure, and oxytocin challenge to mimic cold coagulation and blood stasis syndrome (18). Specifically, as shown in **Figure 1**, rats in the PDG were placed on ice blocks for 20 min daily for 12 consecutive days. Estradiol valerate was administered subcutaneously at 0.4 mg/rat/day from day 2 to day 11, with an increased dose of 0.8 mg/rat on days 1 and 12. On day 12, oxytocin (2 U/rat) was injected intraperitoneally to induce uterine hypercontractility. Control rats received normal saline at 10 mL/kg in place of the corresponding treatments and were not exposed to cold stimulation.

### 2.4 Behavioral pain assessment

Pain responses were evaluated using the writhing test. Following oxytocin administration, rats were observed for 30 min (19). Writhing latency (time to first writhing response) and total number of writhes were recorded according to standardized criteria. Behavioral assessments were performed by investigators blinded to group allocation.

### 2.5 Assessment of uterine and peripheral microcirculation

Rats were anesthetized with isoflurane delivered in 100% oxygen, induced at 4-5% in an induction chamber and maintained at 1-3% via a face mask or nose cone with an oxygen flow rate of 1 L/min (20). Uterine, sublingual, and paw microcirculation were measured using a full-field laser perfusion imager (moorFLPI-2, Moor Instruments, UK). Blood flow was quantified as perfusion units within predefined regions of interest (21). At the end of the experiment, rats were deeply anesthetized with isoflurane and euthanized by exsanguination via abdominal aortic blood collection, after which uterine tissues were harvested.

### 2.6 Biochemical and hormonal measurements

Plasma levels of estradiol (E_2_), progesterone (P), β-endorphin (β-EP), and NO, as well as uterine tissue levels of PGF_2_α, PGE_2_, arginine, and proline, were quantified using commercially available ELISA kits according to the manufacturers’ instructions. NO levels were determined based on nitrate/nitrite concentrations as indicators of total NO production.

### 2.7 Histopathological examination

Uterine tissues were fixed in 4% paraformaldehyde, paraffin-embedded, sectioned (4 μm), and stained with hematoxylin and eosin. Histopathological changes were evaluated using McGuigan’s scoring system by a blinded pathologist (19).

### 2.8 Coagulation function analysis

Plasma coagulation parameters, including prothrombin time (PT), activated partial thromboplastin time (APTT), thrombin time (TT), and fibrinogen (FIB), were measured using a semi-automatic coagulation analyzer (PUN-2048B, Beijing Pulang New Technology Co., Ltd.).

### 2.9 Transcriptomic analysis

Total RNA was extracted from uterine tissue using TRIzol reagent (Life Technologies, California, USA). RNA integrity was confirmed using an Agilent 4150 Bioanalyzer. Strand‐non‐specific RNA‐seq libraries were prepared using the ABclonal mRNA‐seq Lib Prep Kit, with mRNA enriched by oligo(dT) magnetic beads. Libraries were sequenced on the DNBSEQ-T7 platform with paired‐end 150 bp reads, yielding a minimum of 6 Gb raw data per sample. To control for batch effects, samples were randomized across sequencing lanes. Raw reads were subjected to quality control, and reads were discarded if more than 60% of bases had a Phred quality score (Q) ≤ 25 (22). High-quality reads were aligned to the Rattus_norvegicus_Ensembl_104 reference genome using HISAT2(v 2.2.1), achieving an average mapping rate ≥ 90%. Gene expression quantification was performed using FeatureCounts (v 2.0.3). Differential expression analysis was conducted with DESeq2 (v 1.34.0) using the criteria |log_2_FC| ≥ 1 and an FDR < 0.05.

### 2.10 Proteomic analysis

Proteins were extracted from uterine tissues, reduced, alkylated, and digested with Trypsin (enzyme‐to‐protein ratio 1:50) using the FASP method. Peptides were desalted using C18 Cartridges and analyzed by label-free LC-MS/MS on a timsTOF Pro mass spectrometer (Bruker, USA) coupled with an Evosep One system (Evosep, Denmark). Mass spectrometry data were processed using MaxQuant (v 1.6.14) and searched against the Ensembl_Rattus_45936_20220121 protein database supplemented with a common contaminants database. Search parameters included: full tryptic specificity, up to two missed cleavages, fixed modification of carbamidomethyl ^©^, variable modification of oxidation (M), and both peptide and protein false discovery rates (FDR) set to ≤ 1%. Label‐free quantification (LFQ) was performed without normalization. Differentially expressed proteins were identified using the criteria: fold change > 2 or < 0.5 and p < 0.05.

### 2.11 Integrated transcriptomic–proteomic and bioinformatics analysis

Integrated analyses of transcriptomic and proteomic data were conducted using bioinformatics platforms including STRING, Cytoscape, and MetaboAnalyst as appropriate. Pathway and functional enrichment analysis were performed using the Kyoto Encyclopedia of Genes and Genomes (KEGG) and Gene Ontology (GO) databases (23). Overlap and correlation between transcriptomic and proteomic alterations were assessed to identify consistently dysregulated pathways.

### 2.12 Statistical analysis

Data are presented as mean ± standard deviation (SD). All datasets met assumptions of normality and homogeneity of variance as confirmed using built-in analysis modules in GraphPad Prism (version 10.1.2). Comparisons between the two groups were performed using unpaired Student’s t-test. A p-value < 0.05 was considered statistically significant (24).

## 3 Results

### 3.1 Establishment and validation of a rat model of primary dysmenorrhea with cold coagulation and blood stasis syndrome

To establish a reliable animal model of PD accompanied by cold coagulation and blood stasis syndrome, rats were subjected to estradiol priming, oxytocin stimulation, and repeated cold exposure. Behavioral pain responses were first assessed to validate the successful induction of PD.

**Figure 2:**
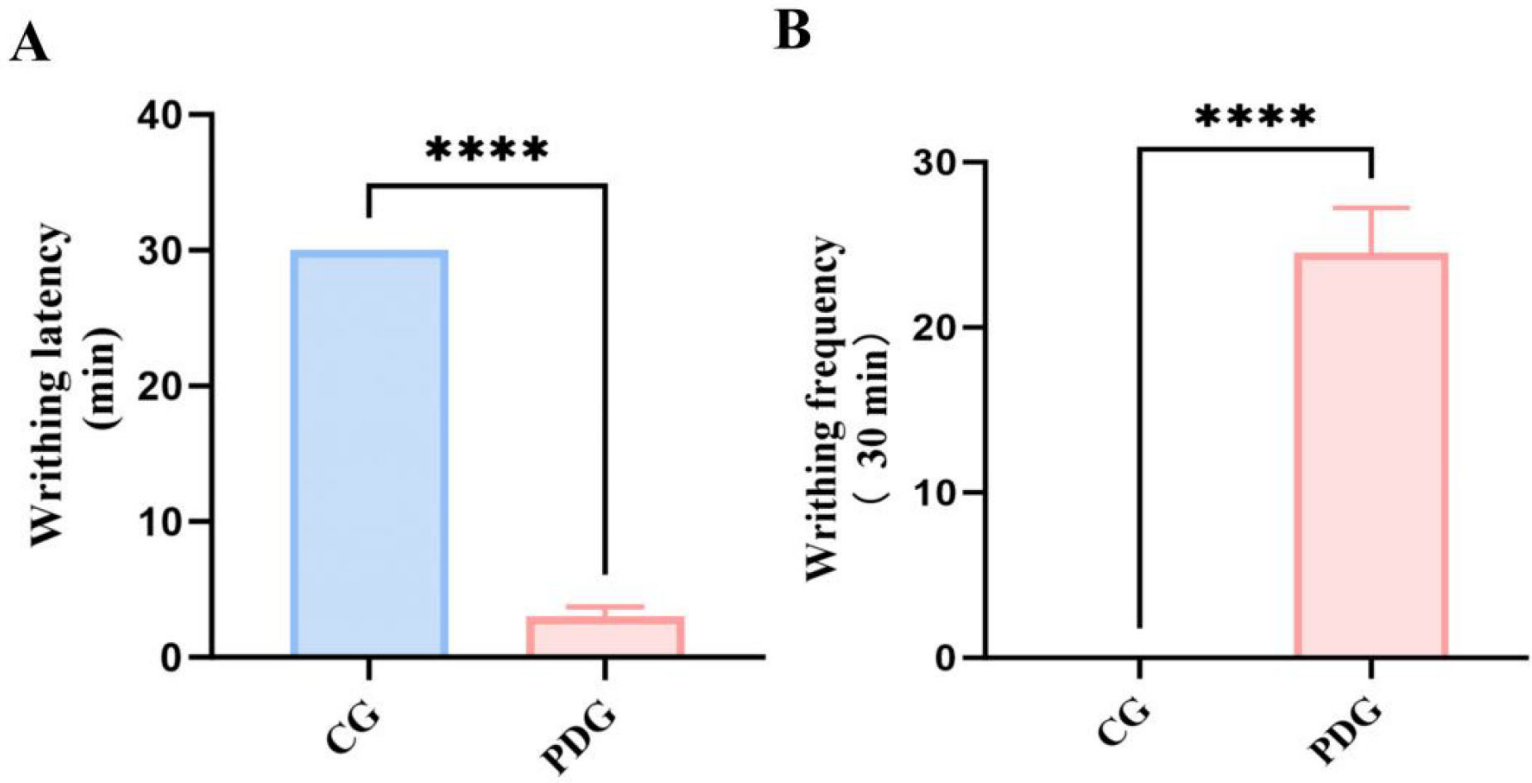
Writhing latency **(A)** and writhing frequency **(B)** in rats of the CG and PDG groups (n = 6). ****P < 0.0001.

**Figure 3:**
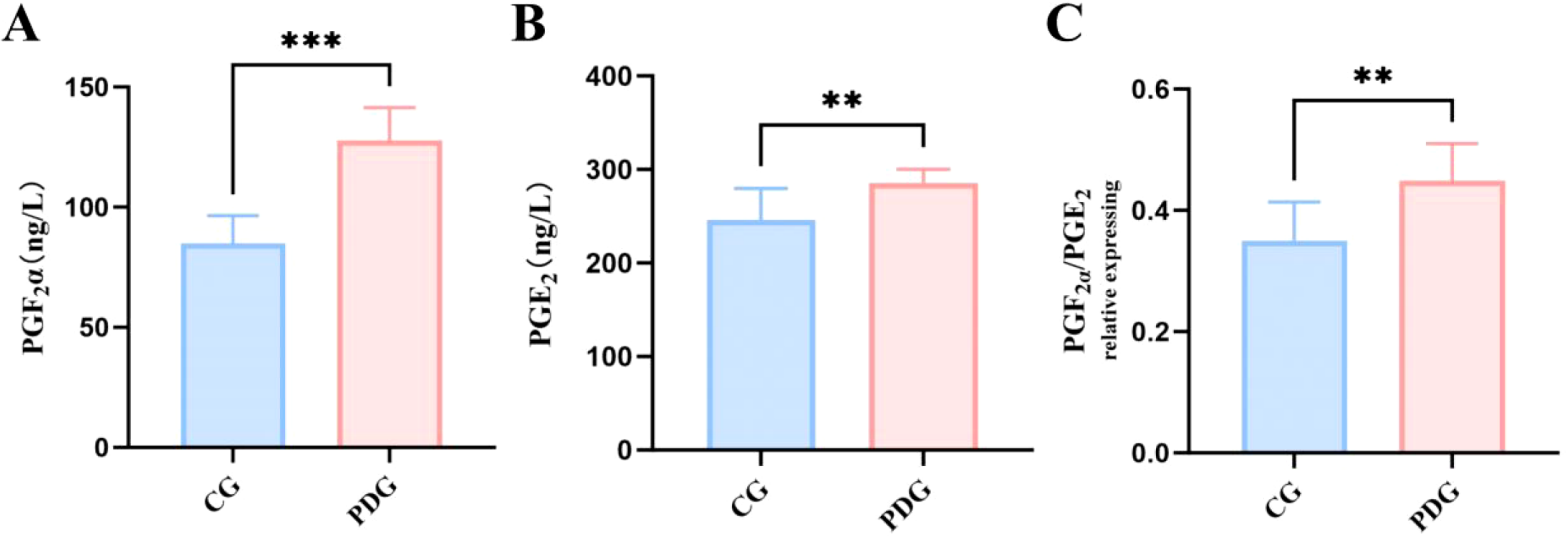
The levels of PGF_2_α **(A)**, PGE_2_ **(B)** and ratio of PGF_2_α/PGE_2_ **(C)** in uterine tissue of PDG group rats (n=6). **P < 0.01, ***P < 0.001.

As shown in **Figure 2**, rats in the PDG group exhibited a dramatically shortened writhing latency (*P* < 0.001) and a markedly increased number of abdominal writhes within 30 min compared with the CG group (*P* < 0.001), indicating enhanced uterine contractility and pain sensitivity following oxytocin challenge. Consistent with behavioral changes, uterine levels of prostaglandins were significantly altered in PD rats. As illustrated in **Figures 3A, B**, both PGF_2_α (*P* < 0.001) and PGE_2_ (*P* < 0.01) contents in uterine tissue were significantly elevated in the PDG compared with the CG. Importantly, the PGF_2_α/PGE_2_ ratio (*P* < 0.01), was also significantly elevated in the PDG suggesting a dysregulated balance of contractile and vasomodulatory prostaglandins rather than a simple absolute increase (**Figure 3C**). This imbalance may contribute to exacerbate abnormal uterine hypercontractility and ischemia, thereby potentially promoting pain generation.

**Figure 4:**
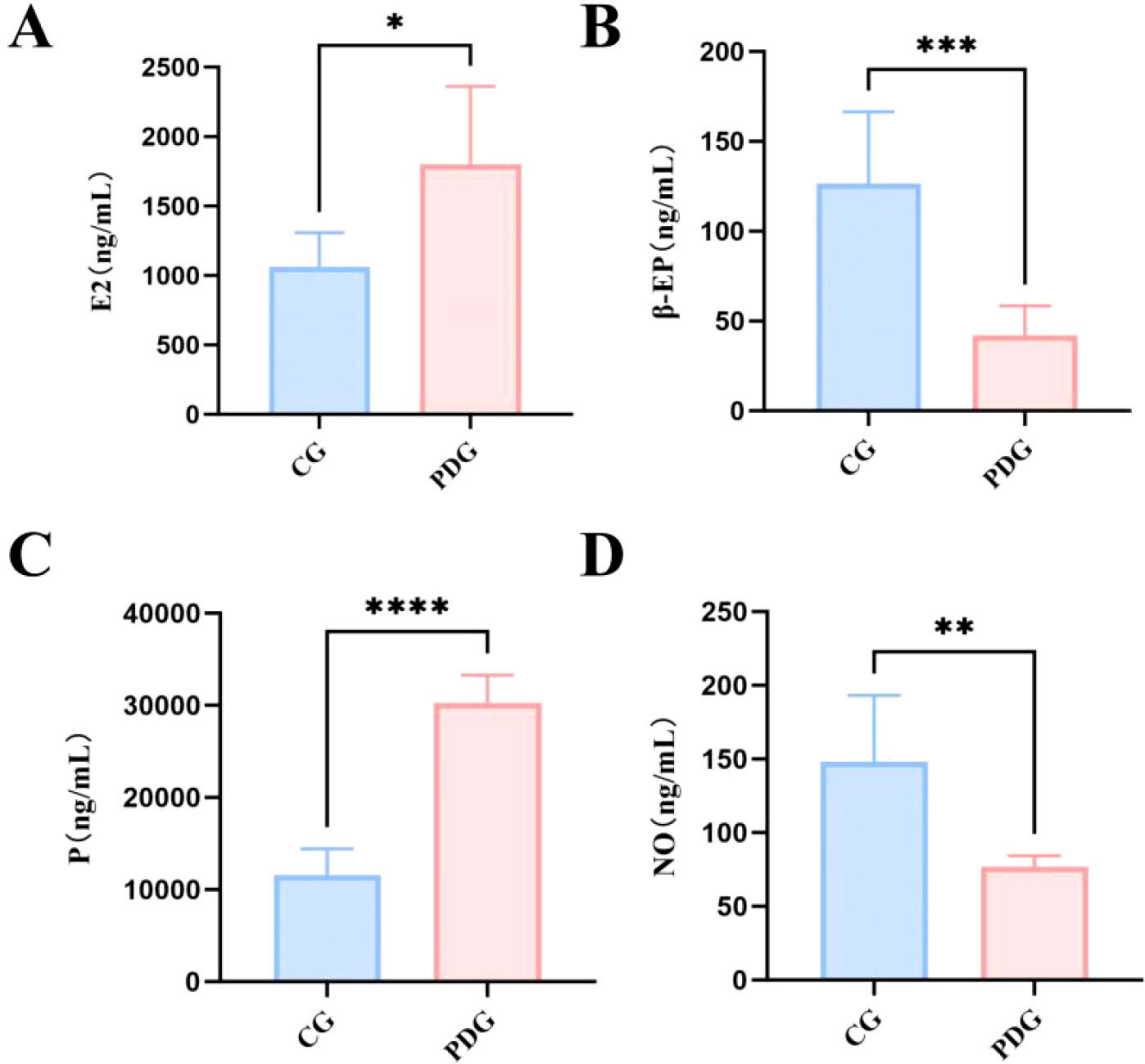
The levels of P **(A)**, E_2_ **(B)**, β-EP **(C)** and NO **(D)** in the plasma of rats in the PDG group (n=6). *P < 0.05, **P < 0.01, ***P < 0.001, ****P < 0.0001.

Plasma levels of hormones and neuromodulators further supported the successful establishment of PD pathology. As shown in **Figure 4**, E_2_ (*P* < 0.05) and P (*P* < 0.0001) levels were significantly elevated in PD rats, reflecting effective hormonal priming. In contrast, β-EP (*P* < 0.001), an endogenous analgesic peptide, and NO (*P* < 0.01), a critical vasodilator, were both significantly decreased in the PDG compared with the CG. These findings are consistent with impaired endogenous analgesic capacity and vascular dysfunction in PD rats.

**Figure 5:**
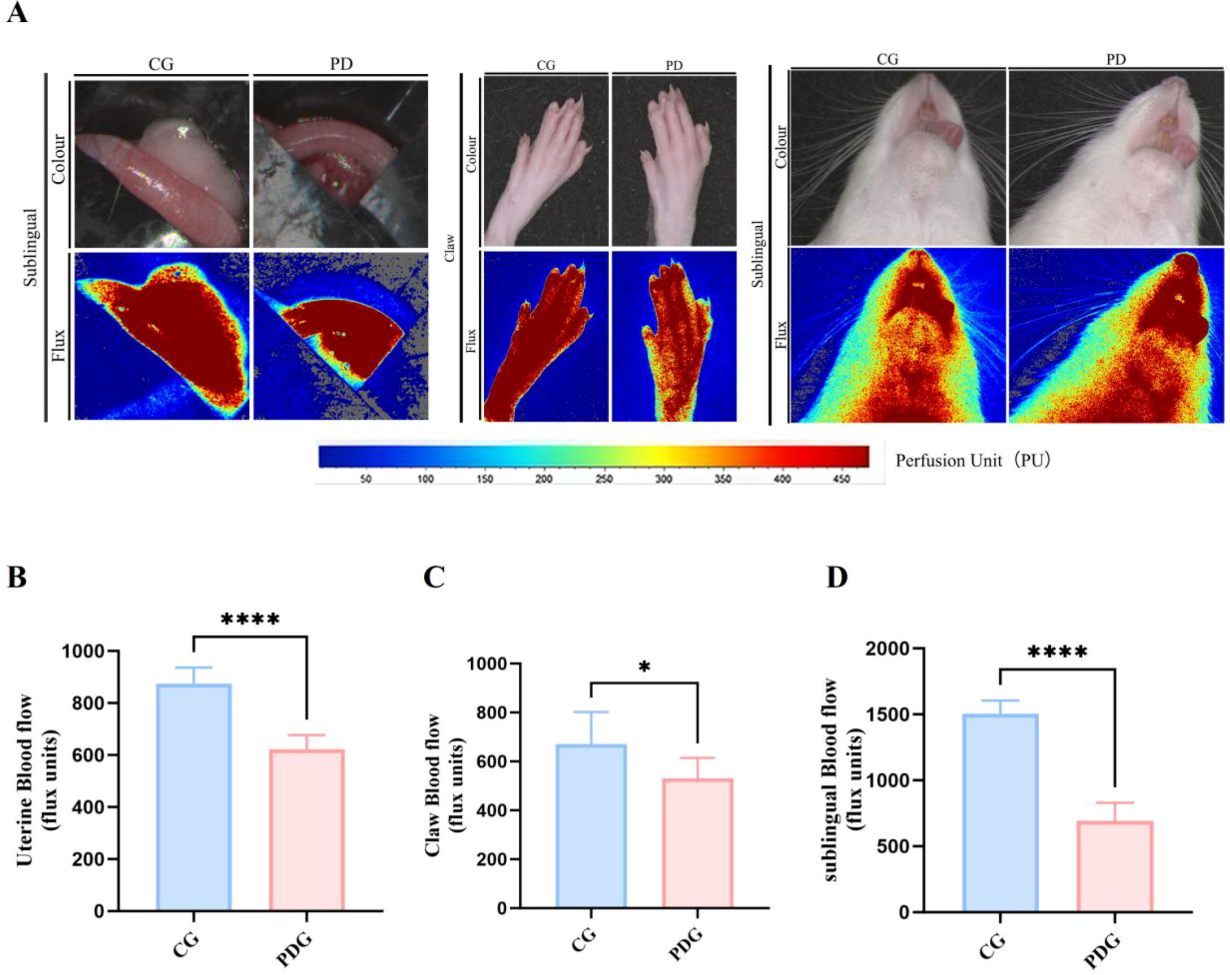
Laser speckle contrast imaging **(A)**; blood flow in the uterus **(B)**, claw **(C)**, and sublingual region **(D)** (n=6). *P < 0.05, ****P < 0.0001.

Microcirculatory alterations were directly visualized using laser speckle contrast imaging (**Figure 5**). Analysis demonstrated that blood flow in the uterine tissue (*P* < 0.0001), claw (*P* < 0.05), and sublingual region (*P* < 0.0001) was significantly reduced in PD rats relative to controls. The pronounced reduction in uterine perfusion is consistent with uterine vasoconstriction and ischemia, which are key features of PD in the context of cold coagulation and blood stasis syndrome.

Collectively, behavioral, biochemical, hormonal, and microcirculatory findings support the successful establishment of a PD rat model that recapitulates key clinical and pathophysiological features of primary dysmenorrhea.

### 3.2 Histopathological alterations in uterine tissue of PD rats

**Figure 6:**
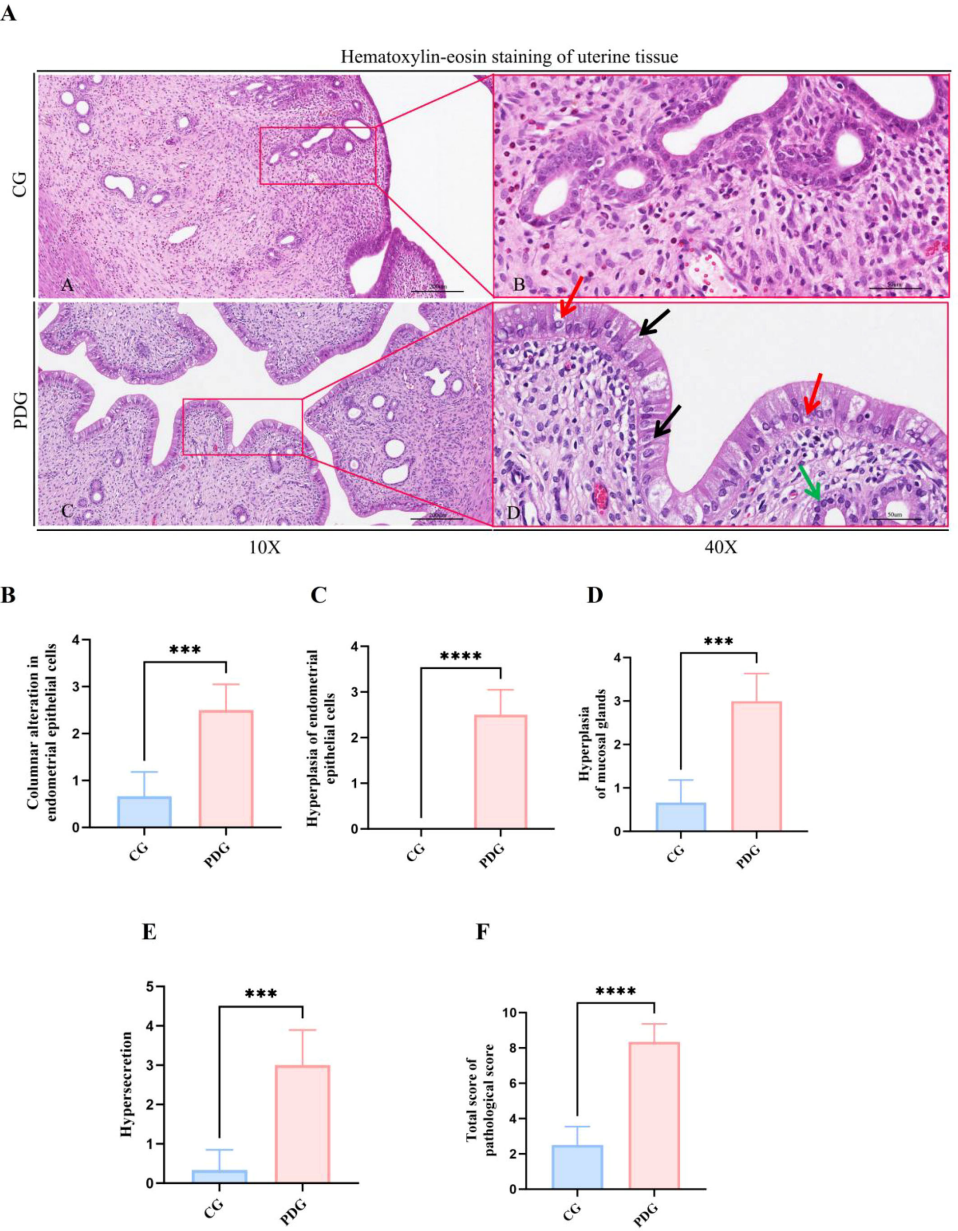
Histopathological analysis of rat uterine tissue. **(A)** Representative histological images of uterine tissue from the CG and PDG groups. The framed regions correspond closely to the magnified views shown on the right. The scale bar for the 10× images is 200 μm, and the scale bar for the 40× images is 50 μm. In the CG group, the uterine tissue structure was intact and clearly organized, with no obvious hyperplasia or inflammatory cell aggregation. In the PDG group, high-columnar transformation of uterine mucosal epithelial cells (black arrows), hyperplasia of uterine mucosal epithelial cells (red arrows), and mucosal gland hyperplasia (green arrows) were observed. Pathological scoring of endometrial epithelial hyperplasia **(B)**, columnar alteration of endometrial epithelial cells **(C)**, mucosal gland hyperplasia **(D)**, hypersecretion **(E)**, and total pathological score **(F)** in the CG and PDG groups (n = 6). ***P < 0.001, ****P < 0.0001.

Histopathological examination further revealed substantial structural changes in the uterus of PD rats. As shown in **Figure 6A**, uterine tissue from the CG exhibited a normal histological architecture, characterized by a single layer of regularly arranged columnar epithelial cells, intact uterine glands, and minimal inflammatory cell infiltration. A sparse infiltration of eosinophils was observed in the lamina propria, and the mucosal layer exhibited normal thickness with no evidence of edema, hemorrhage, or other pathological changes. In contrast, uterine tissue from the PDG displayed pronounced pathological alterations, including hyperplasia of endometrial epithelial cells, hypercolumnar transformation of epithelial cells, marked proliferation of mucosal glands, and dilation of glandular lumens with increased secretory activity. These changes are consistent with hormonally influenced pathological remodeling within the uterine microenvironment.

Quantitative assessment using McGuigan’s pathological scoring system (**Figures 6B–F**) demonstrated that scores for hypercolumnar change (*P* < 0.001), epithelial hyperplasia (*P* < 0.0001), glandular hyperplasia (*P* < 0.001), and increased secretions (*P* < 0.001) were all significantly elevated in PD rats compared with controls (*P* < 0.001). The total pathological score was markedly higher in the PDG (*P* < 0.0001), supporting the presence of uterine hyperplastic and pathological lesions associated with PD.

### 3.3 Coagulation dysfunction in PD rats

**Figure 7:**
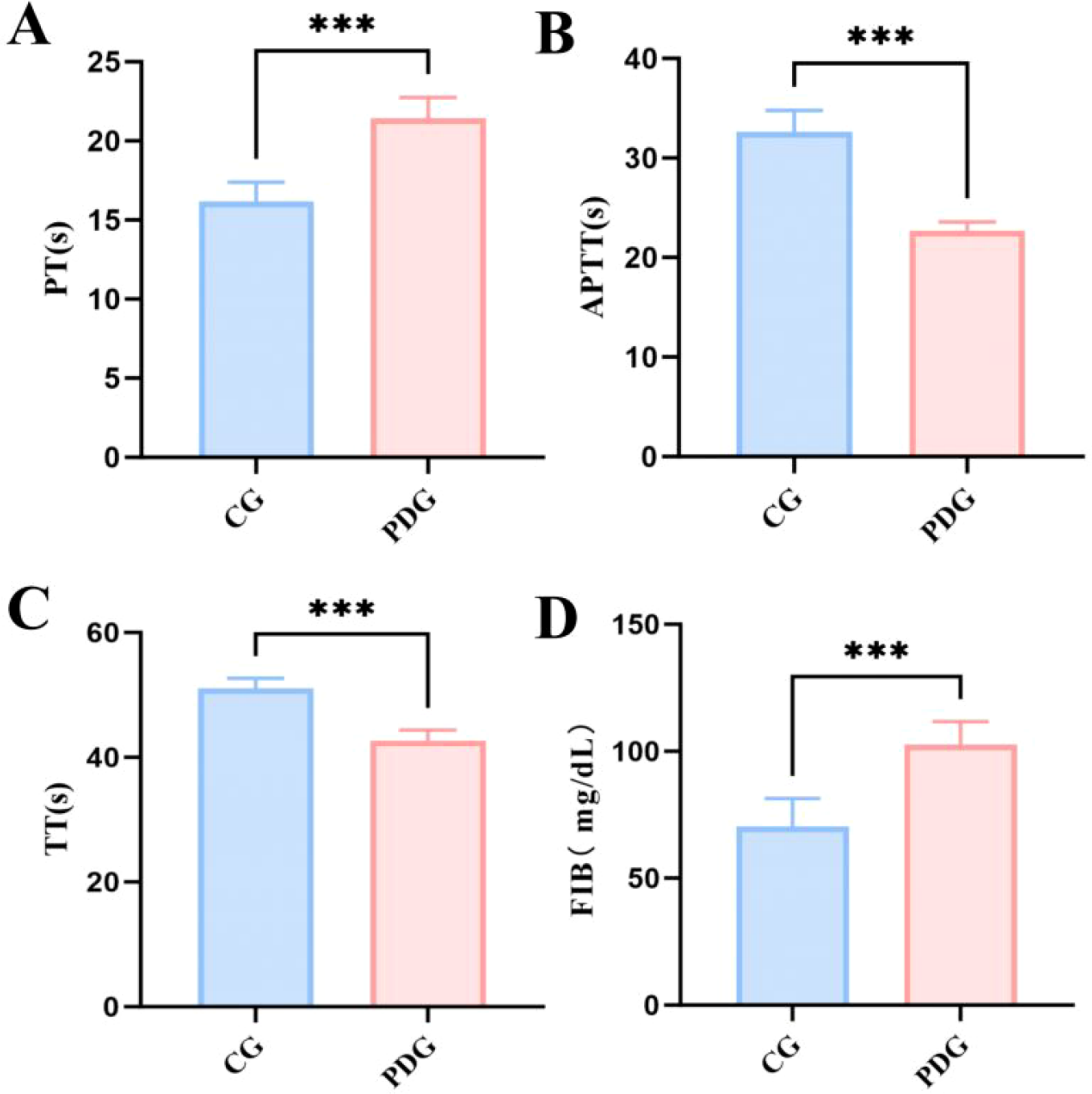
The coagulation indicators in the plasma of PDG group rats: PT **(A)**, APTT **(B)**, TT **(C)**, FIB **(D)** (n=6). ***P < 0.001.

Given that blood stasis is a core pathological feature of PD from both traditional and modern perspectives, plasma coagulation parameters were evaluated. As shown in **Figure 7**, PD rats exhibited a significant decrease in TT and APTT (*P* < 0.001), accompanied by elevated FIB levels and prolonged PT (*P* < 0.001), compared with the CG. These alterations indicate coagulation disturbance in PD rats, which may contribute to impaired uterine microcirculation and tissue ischemia. The coagulation abnormalities observed further support the successful modeling of cold coagulation and blood stasis syndrome in PD.

### 3.4 Transcriptomic profiling reveals widespread gene expression alterations in PD

**Figure 8:**
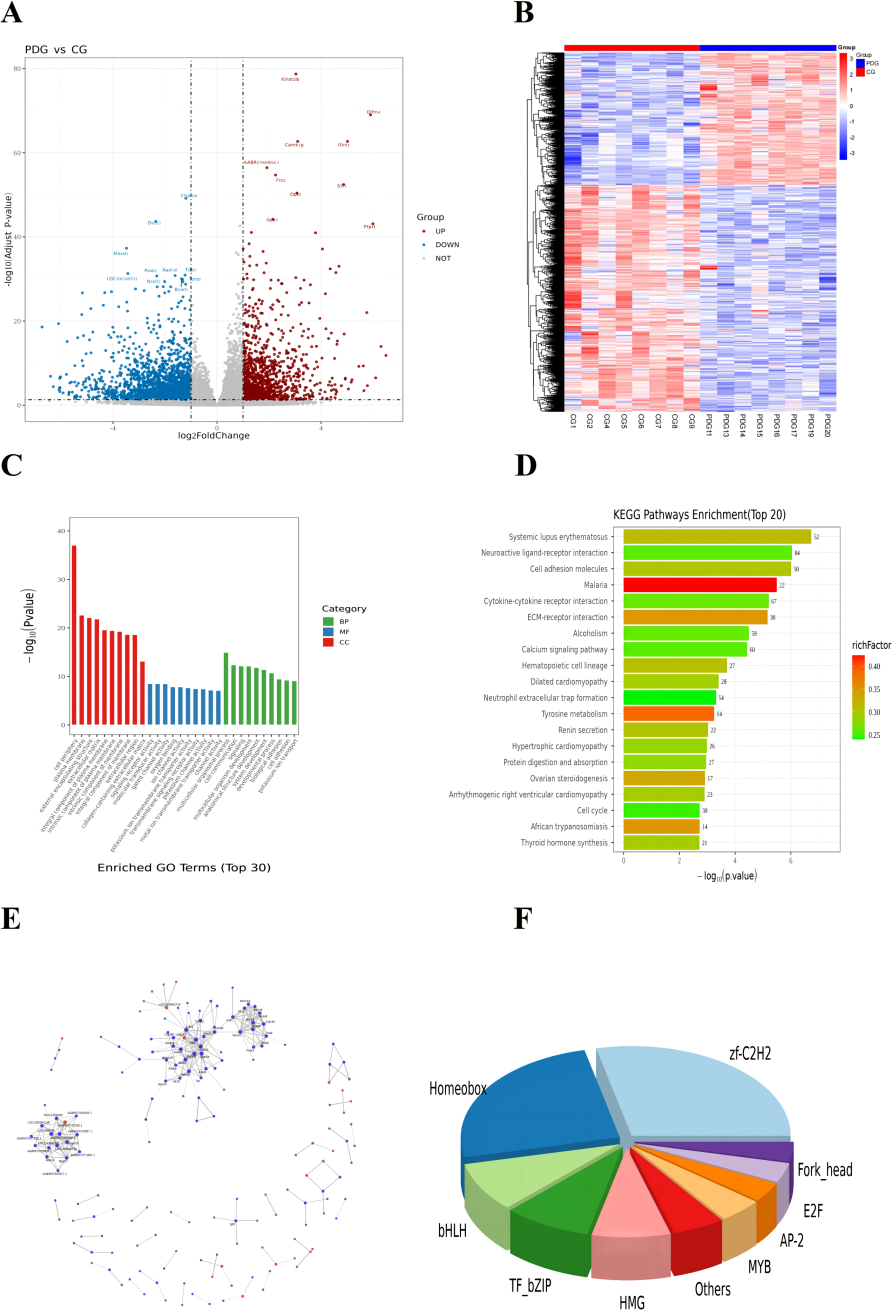
The DEGs in the CG and PDG groups of volcano map **(A)**; Cluster analysis of DEGs in the CG and PDG groups **(B)**; GO enrichment analysis of DEGs **(C)**; KEGG enrichment analysis of DEGs **(D)**; Interaction network of differentially expressed genes (top300) **(E)**; Pie chart of transcription factor families **(F)**.

Transcriptomic analysis was conducted to explore molecular changes associated with PD pathogenesis. Quality control metrics are summarized in **Supplementary Table 1**. A total of 23,295 genes were detected, among which 3,094 were identified as DEGs between the CG and PDG, including 1,142 upregulated and 1,952 downregulated genes (**Figure 8A**).

Hierarchical clustering analysis demonstrated clear segregation between CG and PDG samples (**Figure 8B**), indicating robust transcriptional differences induced by PD modeling. GO enrichment analysis revealed that DEGs were predominantly associated with multicellular organismal processes, molecular transducer activity, and cell periphery components (**Figure 8C**). KEGG pathway enrichment analysis further showed that DEGs were significantly enriched in pathways related to systemic lupus erythematosus, neuroactive ligand-receptor interaction, and cell adhesion molecules (**Figure 8D**), suggesting extensive metabolic and signaling perturbations in PD. Genes are interconnected through a highly complex interaction network, among which Mcm5, Mcm3, Mcm6, Cdc45, Mcm4, and Bub1 exhibit the highest number and greatest complexity of interactions (**Figure 8E**). Our analysis of transcription factor families revealed that the zf-C2H2 and Homeobox families exhibited the most pronounced alterations (**Figure 8F**).

### 3.5 Proteomic analysis identifies dysregulated metabolic and signaling pathways in PD

**Figure 9:**
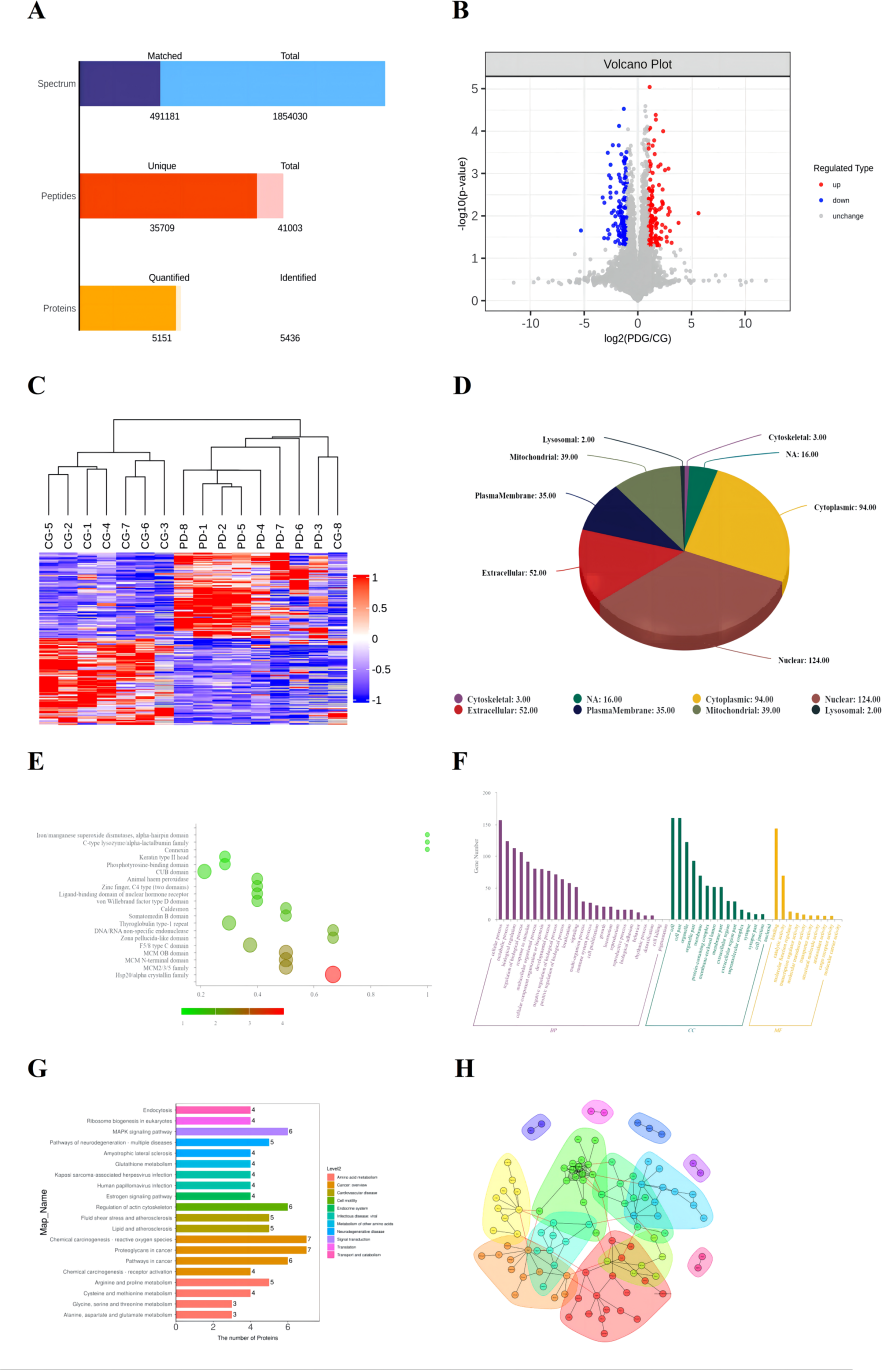
Number of identified proteins **(A)**; Volcano plot of DEPs in the CG and PDG groups **(B)**; Cluster analysis of DEPs in the CG and PDG groups **(C)**; Subcellular localization analysis, with numbers indicating protein counts **(D)**; GO enrichment analysis of DEPs, in which the x-axis represents the rich factor (defined as the ratio of the number of differentially expressed proteins annotated to a given GO term to the total number of identified proteins annotated to that GO term), and bubble color indicates the enrichment significance [−log10(P value)] **(E)**; GO functional classification of DEPs based on the number of annotated proteins in each category **(F)**; KEGG enrichment analysis of DEPs **(G)**; Protein-protein interaction (PPI) network analysis **(H)**.

We successfully matched a total of 491,181 tandem mass spectra from the database, resulting in the identification of 35,709 unique peptides. From these data, we confidently identified 5,346 proteins, with 5,151 quantifiable across more than half of the biological replicates (**Figure 9A**). Differential expression analysis revealed that 285 proteins were significantly altered between the CG and PDG groups, of which 132 were upregulated and 153 were downregulated in PD rats (**Figure 9B**). Consistent with transcriptomic findings, hierarchical clustering clearly distinguished PD samples from controls (**Figure 9C**). Subcellular localization analysis revealed that the majority of differentially expressed proteins were localized in the nucleus and cytoplasm, with a subset also detected in the plasma membrane, mitochondria, and extracellular space (**Figure 9D**). Among the enriched protein domains, the Hsp20/α-crystallin family and the CUB domain were notable, as alterations in these domains may affect relevant protein functions (**Figure 9E**). GO enrichment analysis indicated that differentially expressed proteins (DEPs) were mainly involved in cellular processes, catalytic activity, and cell part organization (**Figure 9F**). KEGG analysis revealed significant enrichment in amino acid metabolism-related pathways, including phenylalanine, tyrosine and tryptophan biosynthesis, arginine biosynthesis, and alanine, aspartate and glutamate metabolism (**Figure 9G**). We also observed that the differentially expressed proteins are highly clustered, forming an intricate interaction network. Based on topological clustering principles, we partitioned the network into distinct clusters, each comprising proteins that likely share similar or related biological functions and act cooperatively to execute specific cellular processes (**Figure 9H**).

### 3.6 Integrated transcriptomic and proteomic analyses highlight arginine and proline metabolism as a central pathway in PD

**Figure 10:**
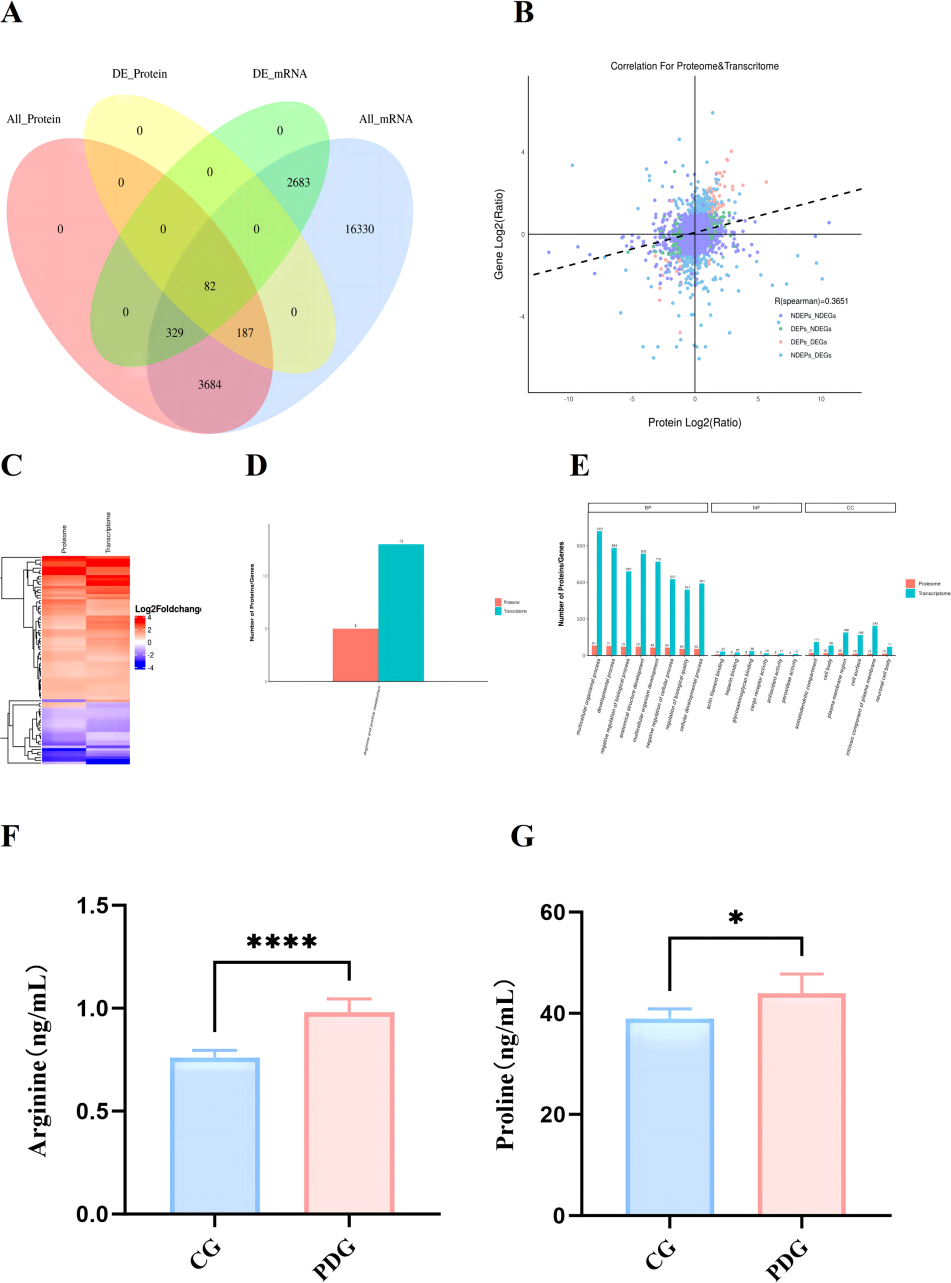
Integrated analysis of DEGs and DEPs **(A)**; Global correlation analysis across samples **(B)**; Cluster analysis of DEGs and DEPs **(C)**; Shared KEGG pathways jointly annotated by the transcriptomic and proteomic datasets, with plotted values indicating the number of differentially expressed molecules annotated to each pathway in the two omics layers **(D)**; Shared GO categories jointly annotated by the transcriptomic and proteomic datasets, with plotted values indicating the number of differentially expressed molecules annotated to each term in the two omics layers **(E)**; Uterine levels of arginine **(F)** and proline **(G)** in the CG and PDG groups (n=6). *P < 0.05; ****P < 0.0001.

To explore coordinated molecular alterations at both transcriptional and translational levels, an integrated analysis of differentially expressed genes (DEGs) and DEPs was performed. Among them, 82 significantly dysregulated gene-protein pairs showed consistent directional changes (**Figure 10A**). Global correlation analysis between all quantified proteins and their corresponding transcripts yielded a Pearson correlation coefficient of 0.3651, indicating modest overall concordance. In contrast, when focusing specifically on significantly differentially expressed genes and proteins, the correlation coefficient markedly increased to 0.8176 (**Figure 10B**), suggesting strong concordance at the level of pronounced molecular alterations. Cluster analysis further revealed that most overlapping genes and proteins exhibited consistent expression trends (**Figure 10C**). Notably, KEGG pathway analysis highlighted arginine and proline metabolism as the only pathway significantly enriched at both transcriptomic and proteomic levels (**Figure 10D**). Integrated GO enrichment analysis showed that both DEGs and DEPs were enriched in multicellular organismal processes, intrinsic components of the plasma membrane, and glycosaminoglycan binding (**Figure 10E**). Targeted measurement further showed that uterine levels of arginine (*P* < 0.0001) and proline (*P* < 0.05) were significantly elevated in PD rats compared with controls (**Figures 10F, G**), suggesting altered arginine utilization and a potential association with reduced NO bioavailability.

## 4 Discussion

PD is a highly prevalent gynecological disorder whose pathogenesis extends well beyond excessive prostaglandin production (25). In the present study, we established a robust rat model of PD with cold coagulation and blood stasis syndrome and performed comprehensive behavioral, biochemical, histopathological, hemodynamic, and integrated transcriptomic and proteomic analyses, together with targeted measurement of arginine and proline. By integrating these findings, we provide a systems-level interpretation of PD pathophysiology and suggest that dysregulation of prostaglandin balance, NO bioavailability, and amino acid metabolism may represent interconnected contributors to uterine ischemia and pain.

### 4.1 PD is characterized by prostaglandin imbalance rather than absolute overproduction

Consistent with classical theories of PD, uterine levels of both PGF_2_α and PGE_2_ were significantly elevated in PD rats. Importantly, however, the PGF_2_α/PGE_2_ ratio was markedly increased, indicating a shift toward a contractile-dominant prostaglandin profile (26). This finding is mechanistically meaningful and aligns closely with the observed phenotypes. PGF_2_α is a potent inducer of uterine smooth muscle contraction and vasoconstriction, whereas PGE_2_ exerts more complex effects, including vasodilation and smooth muscle relaxation depending on receptor subtype activation. An increased PGF_2_α/PGE_2_ ratio therefore favors sustained uterine hypercontractility and reduced perfusion (27). This prostaglandin imbalance may help explain the shortened writhing latency, increased writhing frequency, and pronounced reduction in uterine blood flow observed in PD rats. Importantly, this ratio-based interpretation offers greater explanatory power than absolute prostaglandin levels alone. It suggests that PD may involve not merely prostanoid overproduction, but also a pathological skewing of prostaglandin signaling that may amplify contractile and ischemic stress within the uterus.

### 4.2 Impaired uterine microcirculation represents a central pathological feature of PD

Laser speckle contrast imaging demonstrated significant reductions in uterine, sublingual, and peripheral microcirculation in PD rats, indicating that PD in this model is accompanied by measurable perfusion abnormalities rather than pain behavior alone. Notably, the reduction in uterine blood flow was particularly pronounced, supporting the view that impaired uterine microcirculation may be a central pathological feature of PD. At the same time, the concomitant reductions in sublingual and peripheral blood flow suggest that the observed vascular alterations may not be entirely restricted to the uterus, but could also reflect broader disturbances in microvascular regulation. Nevertheless, because the uterine perfusion deficit was the most prominent and is closely linked to the pain-related phenotype, uterine microcirculatory dysfunction remains a particularly relevant pathological feature in this model. By contrast, the reductions in claw and sublingual blood flow are better interpreted as supportive phenotypic indicators of the cold coagulation and blood stasis model, reflecting broader microcirculatory disturbance consistent with the traditional “cold coagulation and blood stasis” framework rather than constituting the primary PD phenotype itself (28, 29). This microcirculatory impairment is likely multifactorial. The increased PGF_2_α/PGE_2_ ratio observed in this study suggests a shift toward a more contractile and vasoconstrictive prostaglandin profile, which may reduce uterine perfusion through both enhanced myometrial contraction and vascular constriction. Reduced NO bioavailability may further impair local vasodilatory capacity and limit microvascular adaptation. In addition, the observed coagulation abnormalities, including shortened TT and APTT, elevated fibrinogen, and prolonged PT, indicate disturbed coagulation status rather than a uniformly hypercoagulable state; nevertheless, such alterations may still contribute to impaired blood flow regulation and tissue hypoxia (30). Taken together, these findings suggest that microcirculatory dysfunction may serve as an important link between prostaglandin imbalance, vascular dysregulation, and ischemia-related pain sensitization in PD. This interpretation is also broadly consistent with the traditional concept of cold coagulation and blood stasis, in which impaired circulation is considered a key pathological feature.

### 4.3 Role of sex hormones and endogenous analgesic peptides in modulating prostaglandin–NO signaling

P and E_2_ are known to regulate the expression and activity of cyclooxygenase enzymes as well as prostaglandin receptors in uterine tissues (31). Elevated E_2_ has been reported to enhance PTGS2 transcription and increase sensitivity to contractile prostaglandins, while P withdrawal can further exacerbate myometrial excitability. In the present study, altered E_2_ and P levels were observed in PD rats; however, these hormonal changes should be interpreted with caution, because they may partly reflect the estradiol valerate-based induction procedure used to establish the model rather than PD-related endogenous hormonal dysregulation alone. Nevertheless, within the context of this model, the hormonal alterations may still modulate prostaglandin signaling and interact with reduced NO bioavailability, thereby potentially aggravating uterine ischemia and pain (32, 33). β-EP, a key endogenous opioid peptide involved in pain modulation, was also significantly reduced in PD rats (34). Rather than serving as a primary pathogenic factor, the decline in β-EP likely reflects impaired endogenous analgesic capacity under sustained pathological and ischemic stress (35). Reduced NO availability and microcirculatory dysfunction may further compromise central and peripheral opioid signaling, thereby lowering pain thresholds and enhancing nociceptive responses. Taken together, these findings suggest that hormonal dysregulation and endogenous analgesic failure act as secondary modulators that intensify prostaglandin–NO–mediated uterine dysfunction and pain perception in PD.

### 4.4 Reduced NO bioavailability may link vascular dysfunction and pain sensitization

NO is a critical regulator of uterine vascular tone and smooth muscle relaxation. Diminished NO bioavailability may contribute to sustained vasoconstriction, impaired microcirculation, and enhanced uterine contractility (36). The reduction in NO may help explain the amplified pain responses observed in PD rats. Beyond its vascular effects, NO modulates nociceptive signaling by regulating neurotransmitter release and neuronal excitability (37). Therefore, NO deficiency may simultaneously aggravate ischemia and lower pain thresholds, creating a vicious cycle that perpetuates dysmenorrheic pain. Importantly, the decrease in NO despite elevated arginine levels suggests that altered arginine utilization may warrant further consideration in PD.

### 4.5 Integrated transcriptomic and proteomic analyses highlight arginine–proline metabolism as a convergent pathway in PD

Integrated transcriptomic and proteomic analyses identified arginine and proline metabolism as the only pathway consistently enriched at both transcriptional and translational levels. This convergence suggests that dysregulated arginine–proline metabolism may represent a robust molecular feature of PD rather than an isolated downstream change. Given the central role of arginine as the substrate for NOS (12), this pathway-level convergence provides a rationale for further examining the link between altered arginine utilization and reduced NO bioavailability.

### 4.6 Arginine–proline metabolic dysregulation may contribute to impaired NO bioavailability and microcirculatory dysfunction in PD

The accumulation of uterine arginine and proline, together with reduced systemic NO levels, suggests altered arginine utilization in PD. Because arginine is the sole substrate for NOS, elevated arginine in the setting of reduced NO may reflect impaired substrate utilization, possible alterations in NOS activity, or redirection of arginine metabolism toward alternative pathways. Proline metabolism is closely linked to arginine metabolism through shared intermediates and mitochondrial redox regulation. Under ischemic and stress-related conditions, excess proline may disrupt redox homeostasis and potentially favor NOS uncoupling, thereby further reducing NO bioavailability (38, 39). Proteomic findings also indicated changes in proteins related to endothelial function, catalytic activity, and cellular stress responses, which are consistent with impaired NO signaling and reduced vascular adaptability (40). Integrated with the targeted quantification of arginine and proline, these observations support a model in which arginine–proline metabolic dysregulation may contribute to reduced microvascular dilation and uterine ischemia. However, these mechanistic interpretations remain exploratory and require validation in larger independent cohorts and by targeted functional studies.

### 4.7 Inflammation-related mechanisms, hormonal imbalance, and vascular dysfunction may interact to impair NO signaling

The reduction in NO bioavailability in PD is likely multifactorial. Based on previous studies, inflammatory mediators, oxidative stress, hormonal fluctuations, and vascular dysfunction may all influence NOS activity or NO signaling (41). In the present model, these factors may converge on the NO–vascular axis and further contribute to microcirculatory impairment, NO–vascular ischemia and pain.

### 4.8 Integration of molecular, biochemical, and physiological findings supports a systems-level model of PD

**Figure 11:**
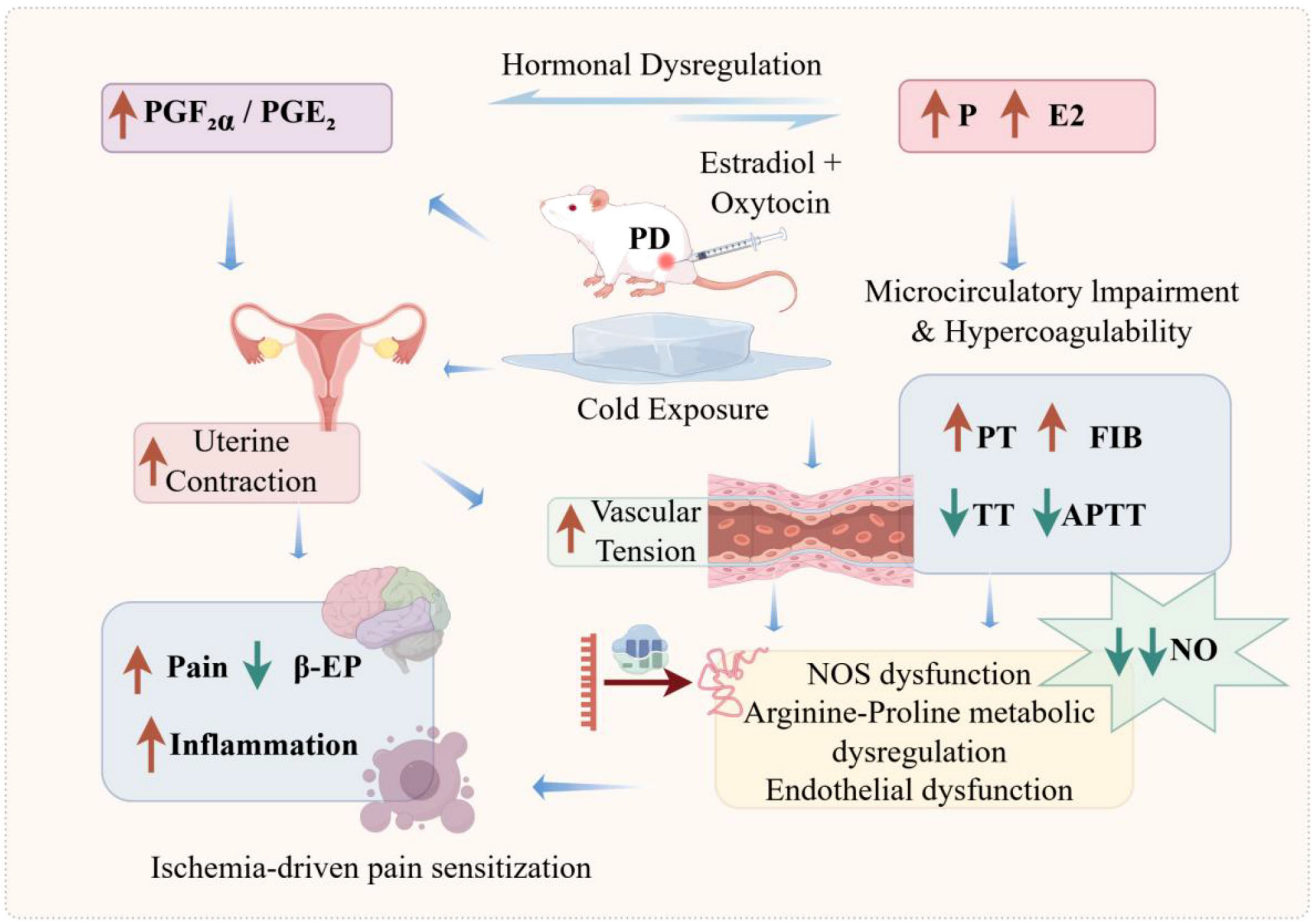
Proposed mechanisms underlying PD (created with Figdraw). Prostaglandin imbalance (↑PGF_2_α/PGE_2_) may promote uterine hypercontractility and vasoconstriction, thereby contributing to impaired uterine microcirculation and coagulation disturbance (PT, APTT, TT, FIB). Reduced perfusion may be associated with altered arginine metabolism and reduced NO bioavailability, which in turn may contribute to vascular dysfunction and uterine ischemia. Hormonal alterations (P and E_2_) may further influence prostaglandin synthesis and uterine contractility. Together, these interconnected events may contribute to ischemia-driven pain sensitization and reduced β-EP within a proposed positive feedback loop that may amplify dysmenorrheic pain.

By integrating behavioral, biochemical, histological, hemodynamic, transcriptomic, and proteomic data, this study supports a systems-level model of PD (**Figure 11**). This model reconciles traditional prostaglandin-centered theories with emerging insights into vascular biology and metabolic regulation, offering a more comprehensive framework for understanding PD.

Previous studies have largely focused on prostaglandin synthesis inhibition as the primary therapeutic strategy for PD. While effective in symptom relief, such approaches do not fully address underlying vascular and metabolic dysfunction. Our findings extend existing knowledge by highlighting the potential relevance of the arginine–NO axis to uterine perfusion and pain. Notably, the integration of transcriptomic and proteomic data strengthens the biological relevance of these findings and reduces the likelihood of spurious associations, distinguishing this study from prior single-omics investigations.

### 4.9 Limitations and future perspectives

Although this study provides evidence supporting the involvement of prostaglandin imbalance, NO deficiency, and arginine–proline metabolic dysregulation in PD, several limitations should be acknowledged. First, the sample size was relatively small, particularly for the transcriptomic and proteomic analyses (n = 6 per group), which may limit statistical power and increase the risk of false discovery in high-throughput datasets. Therefore, the omics findings in this study should be interpreted as exploratory and hypothesis-generating rather than definitive. Second, although integrated transcriptomic and proteomic analyses together with targeted measurement of arginine and proline supported the proposed mechanism, additional validation in larger independent cohorts and by targeted approaches is still needed. Third, direct measurement of NOS isoform activity and oxidative stress markers would further strengthen the mechanistic interpretation. In addition, no formal *a priori* power analysis was performed for sample size determination, which represents another limitation of the current study. Moreover, inflammatory cytokines, immune cell infiltration, and other direct indicators of inflammatory activation were not assessed in this study, which limits a more definitive interpretation of the inflammatory contribution to the observed findings. Future studies with larger sample sizes, formal power calculation, and independent validation are warranted to confirm the robustness of the identified pathways. Furthermore, functional studies targeting the arginine–NO axis, including modulation of NOS activity and pathway-specific intervention experiments, will be important to further clarify causality and therapeutic potential in PD.

## 5 Conclusion

In this study, we established a rat model of primary dysmenorrhea with cold coagulation and blood stasis syndrome and integrated behavioral, biochemical, microcirculatory, histopathological, and transcriptomic-proteomic analyses to explore its underlying pathophysiology. Our findings support a systems-level model in which prostaglandin imbalance, reduced NO bioavailability, coagulation-associated microcirculatory dysfunction, and dysregulated arginine–proline metabolism may jointly contribute to uterine ischemia and pain sensitization. Compared with previous studies that have focused mainly on prostaglandin excess, our work highlights the arginine–NO axis and metabolic-vascular coupling as important components of PD pathophysiology, while also identifying arginine and proline metabolism as a convergent pathway altered at both transcriptomic and proteomic levels. These findings suggest that therapeutic strategies aimed at restoring uterine microcirculation and arginine–NO signaling may complement conventional prostaglandin-centered treatment approaches. Nevertheless, given the limited sample size and the exploratory nature of the transcriptomic and proteomic analyses, further validation in larger cohorts and functional studies is required.

## Data Availability

The raw data supporting the conclusions of this article will be made available by the authors, without undue reservation.
