# Supplementary material for: Integrated transcriptomic and proteomic profiling implicates prostaglandin–nitric oxide network dysregulation in uterine microcirculatory impairment in primary dysmenorrhea
Source: Front Immunol. 2026 May 11;17:1833346. doi: 10.3389/fimmu.2026.1833346 (PMC13199096; doi:10.3389/fimmu.2026.1833346)
Supplement: Supplementary file 1 [file Table1.docx]

**Table S1 The results of sequencing data quality preprocess**

| Sample | Raw_reads | Clean_reads | Clean_bases | Error(%) | Q20(%) | Q30(%) | GC(%) |
| --- | --- | --- | --- | --- | --- | --- | --- |
| CG1 | 67977530 | 67142090 | 10.05G | 0.04 | 97.07 | 93.10 | 49.85 |
| CG2 | 56860412 | 56140344 | 8.4G | 0.04 | 97.01 | 92.93 | 49.68 |
| CG4 | 60653896 | 59846614 | 8.96G | 0.04 | 97.07 | 93.08 | 49.62 |
| CG5 | 45907954 | 45330946 | 6.79G | 0.04 | 97.58 | 94.16 | 49.93 |
| CG6 | 44269834 | 43689332 | 6.54G | 0.04 | 97.02 | 93.00 | 49.95 |
| CG7 | 49561218 | 48925574 | 7.33G | 0.04 | 97.01 | 92.95 | 49.86 |
| CG8 | 60142220 | 59067936 | 8.85G | 0.04 | 96.87 | 92.72 | 49.73 |
| CG9 | 63685292 | 62781060 | 9.4G | 0.04 | 96.99 | 92.90 | 49.65 |
| PDG11 | 57810374 | 56958726 | 8.53G | 0.04 | 96.84 | 92.58 | 50.06 |
| PDG13 | 52328946 | 51418070 | 7.7G | 0.04 | 96.74 | 92.40 | 50.66 |
| PDG14 | 53778624 | 53127404 | 7.95G | 0.04 | 97.12 | 93.12 | 50.50 |
| PDG15 | 57155102 | 56352306 | 8.44G | 0.04 | 96.99 | 92.88 | 50.53 |
| PDG16 | 55755626 | 54997358 | 8.24G | 0.04 | 97.14 | 93.18 | 50.62 |
| PDG17 | 58698634 | 57874734 | 8.67G | 0.04 | 97.05 | 92.99 | 50.22 |
| PDG19 | 52096932 | 51262868 | 7.68G | 0.04 | 97.03 | 92.95 | 50.35 |
| PDG20 | 52224242 | 51592364 | 7.72G | 0.04 | 97.28 | 93.45 | 50.92 |
